# Ecological and Individual‐Level Factors Associated With Hantavirus Cardiopulmonary Syndrome Notifications and in‐Hospital Mortality Among Hospitalized Patients in Chile, 2005–2022

**DOI:** 10.1111/zph.70084

**Published:** 2026-08-16

**Authors:** Benjamín Diethelm‐Varela, Nicolhole Atero, Fernanda Sánchez‐Rodríguez, Constanza Martínez‐Valdebenito, Carolina Henríquez, Francisca Córdova‐Bührle, Marcela Ferrés, Fernando O. Mardones

**Affiliations:** ^1^ Escuela de Gobierno Pontificia Universidad Católica de Chile Santiago Chile; ^2^ SENTINET: Surveillance, Epidemiology and New Technologies for Infectious Emerging Threats Santiago Región Metropolitana Chile; ^3^ One Health Institute & PhD Program in Conservation Medicine, Faculty of Life Sciences Universidad Andres Bello Santiago Chile; ^4^ Departamento de Enfermedades Infecciosas e Inmunología Pediátrica, Escuela de Medicina Pontificia Universidad Católica de Chile Santiago Chile; ^5^ Departamento de Medicina Interna, Escuela de Medicina Pontificia Universidad Católica de Chile Santiago Chile; ^6^ Facultad de Ciencias Veterinarias y Pecuarias (FAVET) Universidad de Chile Santiago Chile; ^7^ Division of Global Agriculture and Food Systems, the Royal (Dick) School of Veterinary Studies The University of Edinburgh Edinburgh UK

**Keywords:** environmental disturbances, forest fires, indigenous populations, *Oligoryzomys longicaudatus*, one health, public health, rodent reservoirs, seasonal trends, surveillance, zoonotic diseases

## Abstract

**Introduction:**

Hantavirus cardiopulmonary syndrome (HCPS) is a severe zoonotic disease caused by *Orthohantavirus andesense* (Andes virus, ANDV), with significant public health burden in Chile. Despite decades of surveillance, knowledge gaps remain regarding the ecological drivers of notification burden and the individual‐level determinants of in‐hospital mortality among hospitalized patients. This study aimed to address both dimensions simultaneously, providing a multilevel characterization of HCPS epidemiology in Chile from 2005 to 2022.

**Methods:**

Two complementary analyses were conducted. First, an ecological study examined district‐level HCPS notifications (2005–2022) using a negative binomial mixed‐effects model, with predictors including male population rate, urbanization proportion, population density, season and burned area due to forest fires during the most recent summer season. Second, an individual‐level analysis of hospitalized HCPS patients (2010–2020) used a logistic mixed‐effects model to identify correlates of in‐hospital mortality among hospitalized patients, including age, sex, indigenous group affiliation, hospitalization duration and number of surgical interventions.

**Results:**

A total of 886 HCPS cases were notified across 187 districts, averaging 49.2 cases per year. Notifications were positively associated with higher male population rates and greater burned area due to forest fires, and negatively associated with urbanization and population density. Among 462 hospitalized patients, the in‐hospital case‐fatality ratio was 19.7%. Older age, female sex and indigenous group affiliation were significantly associated with higher in‐hospital mortality risk among hospitalized patients. Shorter hospitalization duration and greater number of surgical interventions, as correlates of clinical severity and complexity, were also associated with in‐hospital mortality among hospitalized patients.

**Conclusions:**

HCPS notification burden in Chile is shaped by environmental and demographic factors, with forest fire activity representing a novel and actionable ecological risk indicator. In‐hospital mortality among hospitalized patients is disproportionately concentrated in older, female, and indigenous populations, highlighting the need for targeted surveillance, equitable healthcare access and integrated One Health strategies to reduce the burden of HCPS in Chile.

## Impacts

- The present study addressed the epidemiology of hantavirus cardiopulmonary syndrome (HCPS) from both an ecological and individual‐level perspective.
- We found an association between recent forest fires and the risk of notifying new HCPS cases in a district.
- Our results show that HPCS in‐hospital mortalities among HCPS hospitalized patients are higher in female patients, older individuals and patients of indigenous affiliation.

## 1 Introduction

Several members of the Orthohantavirus genus are pathogens of considerable public health concern, causing severe respiratory and renal diseases (Knipe and Howley 2013; Butler and Peters 1994; Vial et al. 2023). In the Americas, infections are caused by *Orthohantavirus andesense* (Andes virus, ANDV), and *Orthohantavirus sinnombreense* (Sin nombre virus, SNV), both associated with the most severe form of infection, hantavirus cardiopulmonary syndrome (HCPS), whereas in Europe and Asia, infections primarily target the kidneys (Butler and Peters 1994). HCPS is a zoonosis caused by several hantavirus species (Centers for Disease Control and Prevention 2021). In North America, most cases are attributed to SNV (Thorp et al. 2023), while in South America, ANDV is one of the most important causative agents (Vial et al. 2023). ANDV transmission to humans occurs mainly through aerosolized secretions of infected rodents, although person‐to‐person transmission has been documented (Ferrés et al. 2007; Martinez‐Valdebenito et al. 2014; Ministerio de Salud 2013). In Chile, the main rodent reservoir is *Oligoryzomys longicaudatus* , the “long‐tailed pigmy rice rat” (Medina et al. 2009; Sotomayor P and Aguilera S 2000; Torres‐Pérez et al. 2019), distributed from the Atacama Desert to the Navarino Islands (Belmar‐Lucero et al. 2009; Palma, Boric‐Bargetto, et al. 2012). However, hantavirus infections in humans have been reported only between the south of the Coquimbo Region and the Aysén Region (Ministerio de Salud 2013). After an incubation period of 7 to 45 days, human infection initially presents with fever, myalgias, headache and in some cases gastrointestinal symptoms. The disease progresses from mild disease to HCPS in more than 50% of cases, characterized by respiratory failure and cardiac compromise, requiring hospitalization and advanced life support. The case‐fatality rate of HCPS is approximately one‐third (Vial et al. 2023; Reyes et al. 2019). Consequently, since 2017, clinical guidelines in Chile mandate prompt hospitalization and immediate transfer to a tertiary hospital with intensive care units and extracorporeal membrane oxygenation (ECMO) availability for all suspected and confirmed hantavirus infection cases (Departamento de Epidemiología. Ministerio de Salud. Gobierno de Chile 2021). Due to its severe presentation and burden on public health systems, HCPS is a mandatory notifiable disease (Sotomayor P and Aguilera S 2000; Reyes et al. 2019). Since 1995, the Chilean Ministry of Health (MINSAL) has maintained a public database of hantavirus infection notifications, listing all known cases with anonymized demographic information. Additionally, the Chilean Department of Health Statistics and Information (DEIS) maintains a public database of all HCPS hospitalizations, which includes all disease‐related in‐hospital mortalities. Despite several decades of research (Torres‐Pérez et al. 2019; Armién et al. 2023; Täger et al. 2003; Gutiérrez‐Jara et al. 2023; Fritz et al. 2023), knowledge gaps concerning the determinants of HCPS remain, both in terms of factors driving hantavirus infection caseloads and risk factors for HCPS fatality. At the ecological level, several phenomena have been proposed as potential drivers of epidemic pulses, including unusual rainfall patterns, forest fires and cyclic increases in rodent populations driven by the overgrowth of wild plants that serve as a food source for the rodents (Gutiérrez‐Jara et al. 2023; Douglas et al. 2021; Holz and Palma 2012; Jaksic and Lima 2003). Previous research suggests that wildfires may significantly influence zoonotic diseases by disrupting animal populations and causing forced displacement of species that may act as vectors or reservoirs of human pathogens (Bonilla‐Aldana et al. 2019; Pascoe et al. 2020; Ecke et al. 2019). At the patient level, the role of gender in the disease has been extensively discussed, as most hantavirus cases occur in males (Fritz et al. 2023); however, whether this is due to biological mechanisms or higher occupational hazards in male‐dominated jobs remains unclear (Riccò et al. 2021; Departamento de Epidemiología. Subsecretaría de Salud Pública. Ministerio de Salud. Gobierno de Chile 2021). Conversely, descriptive studies have identified higher case‐fatality rates among female patients, though formal statistical studies confirming this observation are lacking (Departamento de Epidemiología. Subsecretaría de Salud Pública. Ministerio de Salud. Gobierno de Chile 2021). Important gaps remain in our understanding of HCPS at several stages of the disease pathway, both at the ecological level in terms of which drivers explain case incidence, and at the individual level regarding factors that may account for mortality risk. Therefore, we decided to conduct a two‐pronged study, probing into both the ecological and individual components of the disease. This research thus aims to characterize the epidemiology of hantavirus infection at both ecological and individual levels. Ecologically, we examine the drivers of caseloads at the district level, as cases are reported using the district as the smallest spatial administrative division. Individually, we identify factors associated with ANDV infection‐related in‐hospital mortality risk in the hospitalized population.

## 2 Materials and Methods

### 2.1 Study Design

This study used two distinct approaches to analyse hantavirus epidemiology: (1) an ecological study to identify variables associated with hantavirus notifications (e.g., cases) and (2) an individual‐level analysis to examine risk factors for HCPS in‐hospital mortality among hospitalized patients. Generalized mixed‐effects regression models were employed for both analyses, utilizing distinct probability families for the respective datasets.

### 2.2 Data Sources

Nationwide hantavirus notifications were retrieved from the MINSAL database (Ministerio de Salud 2018), which has collected all notifications since 1995. The dataset includes the reporting date, district of residence and diagnosis (coded as ICD‐10: B33.4). To ensure consistency, the study period began in 2005, when notification protocols were standardized. Each case notification was assigned to a district, which served as the spatial unit for analysis. District‐level ecological predictors, such as population density (inhabitants/km^2^), urbanization proportion (proportion of urban residences) and male‐to‐female ratio, were derived from the 2021 census projection data (Instituto Nacional de Estadísticas 2021). Additionally, annual burned area data (km^2^) due to forest fires were sourced from the National Forestry Corporation (CONAF). For individual‐level in‐hospital mortality analyses, data on hospitalized patients were obtained from the DEIS database, encompassing all hospitalizations from 2010 to 2020. This dataset included anonymized patient IDs, hospital names, diagnoses (ICD‐10: B33.4), length of hospital stay, discharge dates, patient ages, genders, indigenous group affiliation, number of surgical interventions and survival outcomes.

### 2.3 Descriptive and Time Series Analysis

Descriptive analyses characterized hantavirus case notifications (2005–2022) and hospitalizations (2010–2020). For notifications, variables included calendar year, season, percentage of district surface burned by forest fires, urbanization proportion, population density and male population rate per 100,000 inhabitants. Data were stratified by urbanization level (urban, rural and mixed). Differences in proportions for categorical variables were assessed using chi‐squared tests, while one‐way ANOVA was used for continuous variables. For hospitalizations, variables included sex, age, hospitalization duration, indigenous group affiliation, pre‐ or post‐2017 (guideline update), number of surgical interventions and survival status. Differences between survivors and in‐hospital mortalities were analysed using chi‐squared tests (categorical variables) and one‐way ANOVA (continuous variables). Time series analyses were conducted using the Augmented Dickey‐Fuller test to assess stationarity. An additive time series model decomposed seasonal, trend and random components. Seasonal Mann‐Kendall and Pettitt's tests detected trends and change points. Detailed results of the time series analysis are provided in the Supporting Information.

### 2.4 Regression Modelling

A negative binomial mixed‐effects model was used to analyse district‐level hantavirus notifications, accounting for overdispersion and excess zeroes. Predictors included male population rate, burned area during the most recent summer season due to forest fires, year, population density, urbanization proportion and season. Provinces were included as random effects to address spatial non‐independence. Our rationale for selecting the most recent summer season as the exposure window for assessing the risk attributed to burned areas during forest fires is that the overwhelming majority of forest fires occur in summer throughout the studied regions, and only a lag structure of the most recent summer season displayed any significant association with the response variable, so we did not consider other lag periods. A logistic mixed‐effects model analysed HCPS in‐hospital mortality risk in hospitalized patients. Risk predictors included age, indigenous group affiliation and sex. The number of surgical interventions and hospitalization duration were also included; however, because both variables emerge during the course of hospitalization rather than at admission, they are best understood as correlates of in‐hospital mortality reflecting disease severity and clinical complexity, rather than as baseline prognostic factors. Hospitals were treated as random effects. Backward selection based on the Akaike Information Criterion (AIC) identified the final models. Predictor coefficients were exponentiated to derive relative risks (RR) and odds ratios (OR). Collinearity was assessed using Pearson's correlation. Model coefficients, standard errors, statistics, *p*‐values and confidence intervals are provided in the Supporting Information.

### 2.5 Statistical Analysis and Data Visualization

All analyses were performed using R version 4.0 with relevant packages for time series, trend detection and mixed‐effects modelling (R Core Team 2023). Data visualization was conducted using QGIS for spatial analyses and R packages for graphical representation.

## 3 Results

Between 2005 and 2022, 886 hantavirus cases were reported across 187 districts in Chile, accounting for 54.2% of all districts (Figure 1). The average annual incidence rate was 49.2 cases per year, with a rate of 4.8 cases per 100,000 inhabitants per year (95% CI: 4.5–5.1). Notifications were highest in the Los Lagos and Aysén regions, which reported 66.5% of all cases. Most notifications (91.4%) occurred in mixed urbanization districts (*p* < 0.001), with a significantly higher number of cases reported in areas where the rodent reservoir is present. Notification rates peaked during the summer season, with a statistically significant seasonal trend (*p* < 0.001) (Table 1).

**FIGURE 1:**
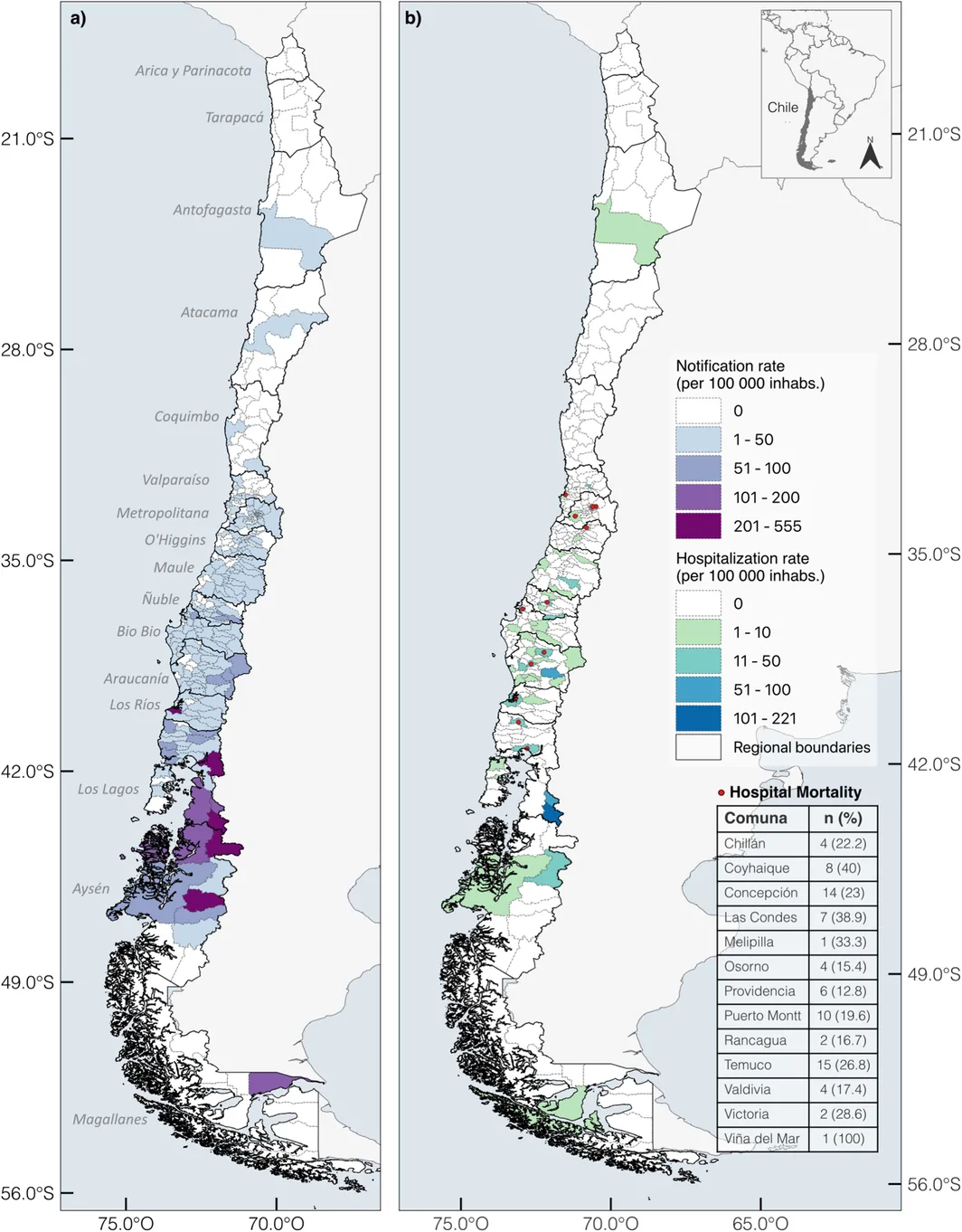
District‐level distribution of cumulative HCPS case notification rates (a) and cumulative HCPS hospitalization rates (b) in Chile. Panel A covers the 2005–2022 notification period; Panel B covers the 2010–2020 hospitalization period. All recorded HCPS events throughout the indicated periods are included. The map was produced using QGIS v3.34. The shape file of the base layer is freely available from the Chilean National Statistics Institute (https://www.ine.gob.cl/herramientas/portal‐de‐mapas/geodatos‐abiertos).

**TABLE 1:** Characteristics of the districts where HCPS was notified throughout the 2005–2022 period, stratified by urbanization level (urban, rural and mixed). Variables include number of notifications, seasonal case distribution, annual case counts, burned surface area due to forest fires during the most recent summer season, population density and male population rate. Differences in proportions for categorical variables were assessed using chi‐squared tests; one‐way ANOVA was used for continuous variables.

Variable	All districts	Urban districts	Rural districts	Mixed urbanization districts	Statistic	*p*
Number of notifications (cases, %)	886	47 (5.3)	29 (3.3)	810 (91.4)	=34.4	< 0.001
Cases per season (mean, SD)	Summer: 0 (0.2) Fall: 0 (0.1) Winter: 0 (0.1) Spring: 0 (0.1)	Summer: 0 (0.1) Fall: 0 (0.1) Winter: 0 (0) Spring: 0 (0.1)	Summer: 0 (0.1) Fall: 0 (0.1) Winter: 0 (0.1) Spring: 0 (0.1)	Summer: 0 (0.2) Fall: 0 (0.1) Winter: 0 (0.1) Spring: 0 (0.1)	F (All) = 60.6 F (Urban) = 16.7 F (Rural) = 1.1 F (Mixed) = 53.62	< 0.001 < 0.001 0.4 < 0.001
Cases per year (mean, SD)	49.2 (15.6)	2.6 (2.3)	1.6 (1.5)	45 (14.9)	*F* = 103.4	< 0.001
Burned surface in forest fires during the most recent summer season as a proportion of the district total area (mean, SD)	0 (0)	5.062524e‐05 (0)	0.000221197 (0)	0.0002975813 (0)	*F* = 30.3	< 0.001
Population density of the district (mean, SD)	1086.8 (3023.6)	9388.37 (3816.6)	3.9625 (10)	217.8471 (699.4)	*F* = 122612.2	< 0.001
Males per 100,000 inhabitants (mean, SD)	50399.6 (4626.1)	48324.29 (1382.7)	62385.14 (12498)	49829.25 (2020.2)	*F* = 21476.9	< 0.001

In the hospitalization dataset (2010–2020), 462 patients were hospitalized for HCPS, with a case‐fatality ratio of 19.7%. Males comprised 73.2% of hospitalized cases, while females accounted for 26.8%. In‐hospital mortality risk was higher among older patients (mean age: 43.8 years) compared to survivors (mean age: 34.3 years, *p* < 0.001). Indigenous group affiliation was reported in 18.8% of cases and was associated with a higher case‐fatality ratio (28% vs. 18%, *p* = 0.06). In‐hospital mortalities were also associated with shorter hospitalization durations (5.1 days for in‐hospital mortalities vs. 12.6 days for survivors, *p* < 0.001) (Table 2).

**TABLE 2:** Demographic and clinical characteristics of patients hospitalized for HCPS during the 2010–2020 period, stratified by survival outcome. Variables include sex, indigenous group affiliation, hospitalization timing relative to the 2017 guideline update, age, hospitalization duration, number of surgical interventions and total number of hospitalizations. Differences between survivors and in‐hospital mortalities were analysed using chi‐squared tests for categorical variables and one‐way ANOVA for continuous variables.

Variable	Total hospitalizations	Survived	Died	Statistic	*p*
Number of hospitalizations (cases, %)	462	370 (80.1)	91 (19.7)	=168.5	< 0.001
Sex (cases, %)	Male: 338 (73.2) Female: 124 (26.84)	Male: 273 (80.77) Female: 97 (78.2)	Male: 65 (19.2) Female: 26 (21)	=0.1	0.75
Member of an indigenous group (cases, %)	Belong: 87 (18.8) Do not belong: 374 (81) Not reported: 1 (0.2)	Belong: 63 (72.4) Do not belong: 306 (81.8) Not reported: 1 (100)	Belong: 24 (27.6) Do not belong: 67 (17.9) Not reported: 0 (0)	=3.5	0.06
Hospitalization before or after the 2017 guidelines were implemented (cases, %)	After: 171 (37) Before: 291 (63)	After: 140 (81.9) Before: 230 (79)	After: 30 (17.5) Before: 61 (21)	=0.5	0.5
Age (years) (mean, SD)	36.2 (17.4)	34.31 (16.9)	43.8 (17.3)	*F* = 22.7	< 0.001
Hospitalization time (days) (mean, SD)	11.1 (12.8)	12.6 (12.5)	5.1 (12.4)	*F* = 26.9	< 0.001
Number of surgical interventions (mean, SD)	0.1 (0.3)	0.1 (0.3)	0.2 (0.4)	*F* = 11.1	< 0.001
Total number of hospitalizations (mean, SD)	1.2 (0.5)	1.2 (0.5)	1.2 (0.4)	*F* = 2.6	0.11

Time series analysis of hantavirus notifications revealed significant seasonal peaks during the summer months (Figures S1, S2 and S5). The Mann‐Kendall test confirmed a declining trend in annual notifications over time (*p* < 0.05) (See Supporting Information). Similarly, HCPS hospitalizations demonstrated seasonal trends aligned with notification data, with peaks in summer months and a slight decline in recent years (Figures S3, S4 and S6). The overall time series of notifications and hospitalizations is presented in Figure 2, with relevant natural disasters annotated.

**FIGURE 2:**
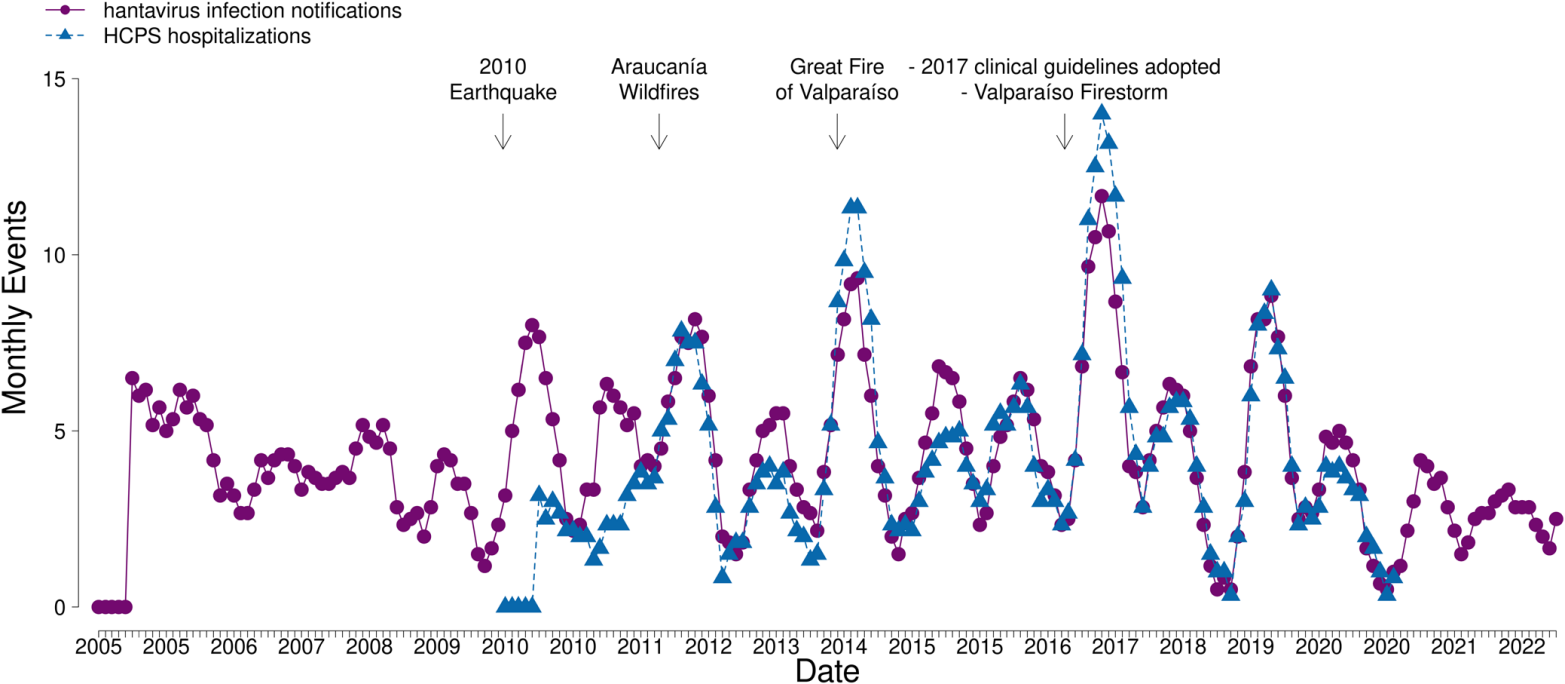
Semestral rolling average of notified HCPS cases and HCPS hospitalizations in Chile across the study period, with relevant natural disasters annotated.

The negative binomial mixed‐effects model identified significant predictors for hantavirus notifications (Figure 3). Higher male population rates (RR = 1.41, 95% CI: 1.11–1.80) and burned area due to forest fires (RR = 1.14, 95% CI: 1.11–1.16) were associated with increased notification risk. Conversely, urbanization proportion (RR = 0.56, 95% CI: 0.50–0.64) and population density (RR = 0.80, 95% CI: 0.68–0.94) were negatively associated with notification risk. Detailed model parameters are provided in Table S1.

**FIGURE 3:**
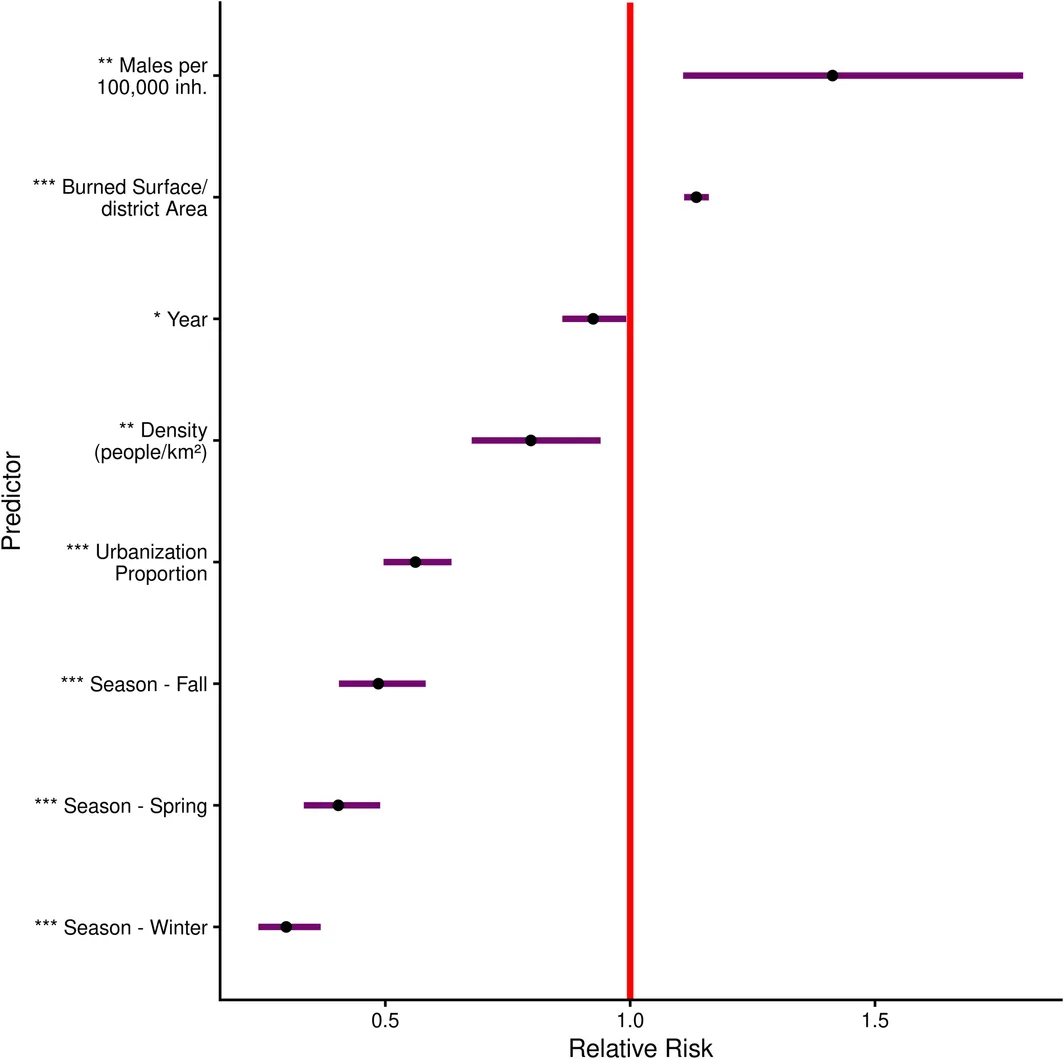
Forest plot displaying the relative risks (RR) and 95% confidence intervals of the predictors included in the negative binomial mixed‐effects regression model for monthly district‐level HCPS notification risk. Reference category for season: Summer. AIC of the null model: 8713.2; AIC of the final model: 8311.0.

The logistic mixed‐effects model identified significant predictors for HCPS in‐hospital mortality risk among hospitalized patients (Figure 4). Regarding prognostic factors, increased age (OR = 2.18, 95% CI: 1.62–2.93), indigenous group affiliation (OR = 2.08, 95% CI: 1.01–4.27) and female sex (OR = 2.03, 95% CI: 1.08–3.82) were associated with higher in‐hospital mortality risk. Regarding intermediate factors regarded as correlates of in‐hospital mortality risk, longer hospitalization duration was associated with reduced in‐hospital mortality risk (OR = 0.13, 95% CI: 0.07–0.24). Surgical interventions increased in‐hospital mortality risk (OR = 2.29, 95% CI: 1.71–3.06). Detailed model parameters are provided in Table S2.

**FIGURE 4:**
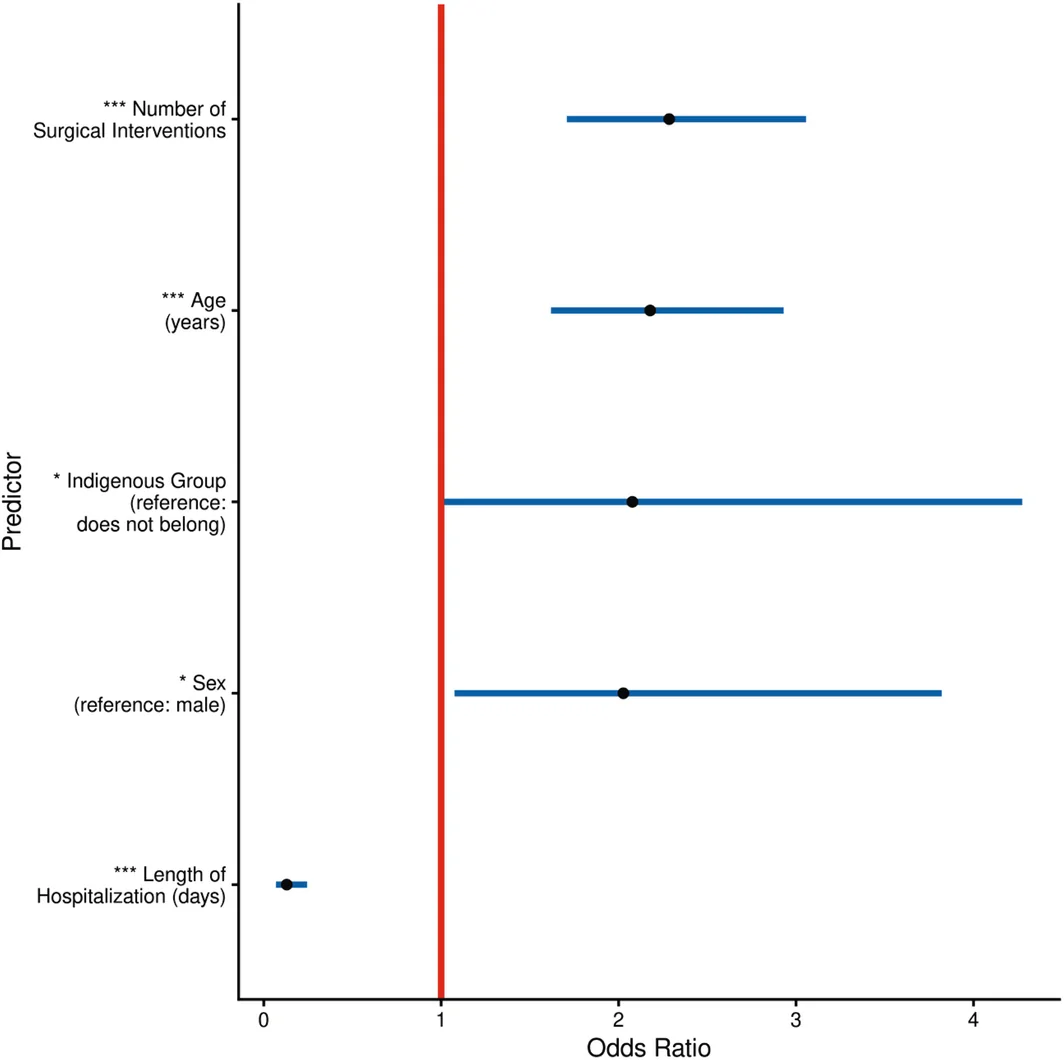
Forest plot displaying the odds ratios (OR) and 95% confidence intervals of the predictors included in the logistic mixed‐effects regression model for in‐hospital mortality risk among hospitalized HCPS patients. AIC of the null model: 458.3; AIC of the final model: 356.1.

## 4 Discussion

This study provides significant insights into the epidemiology of hantavirus in Chile by analysing both ecological and individual‐level risk factors. The rationale behind our decision to conduct both studies stems from the current gaps in our understanding of the complete disease pathway. At the ecological level, knowledge remains limited on the spatial and temporal drivers that influence notification burden. Then, as cases arise, individual considerations take relevance, and here our knowledge is also limited, especially regarding the drivers for mortality in the subset of the population that becomes sufficiently ill to become hospitalized. Therefore, the present study provides contributions to our understanding at both levels of the disease pathway. Our findings reaffirm the seasonal nature of hantavirus infections, with cases peaking in the summer months (Nsoesie et al. 2014; Palma, Polop, et al. 2012). This trend aligns with increased outdoor activities, such as camping and farming, which heighten human exposure to rodent reservoirs ( *Oligoryzomys longicaudatus* ) and their excreta (Butler and Peters 1994; Vial et al. 2023). These results are consistent with previous research linking zoonotic disease dynamics to human‐environment interactions (Torres‐Pérez et al. 2019). The spatial clustering of cases in southern regions, especially Los Lagos and Aysén, reflects the endemic distribution of *O. longicaudatus* (Butler and Peters 1994; Sotomayor P and Aguilera S 2000). The strong association between forest fires and increased hantavirus notifications underscores the potential for environmental disturbances to exacerbate zoonotic disease risks. Wildfires disrupt rodent habitats, increase rodent migration and enhance human exposure (Pascoe et al. 2020; Ecke et al. 2019). Our findings corroborate earlier studies emphasizing the importance of environmental factors in zoonotic disease emergence (Torres‐Pérez et al. 2019; Douglas et al. 2021). Demographic factors such as age, sex and indigenous group affiliation emerged as significant predictors of HCPS fatality. Older patients were at a higher risk of fatal outcomes, potentially due to comorbidities and decreased physiological resilience (Belmar‐Lucero et al. 2009). It should be noted that these results differ from those reported by Thorp et al., who clinically characterized HCPS cases in the United States over 26 years, found a case‐fatality ratio of 35.4% and identified a higher mortality risk in children than in adults (Thorp et al. 2023). These differences may partly reflect differential virulence between ANDV and SNV, but may also be explained, as Thorp and colleagues hypothesize, by the nonspecific clinical presentation of HCPS in children leading to delayed diagnosis—a pattern consistent with findings from Chilean paediatric series, where fever, respiratory distress and gastrointestinal symptoms constituted the main clinical triad and the overall fatality rate reached 36.6% (Ferrés et al. 2010). The higher in‐hospital fatality rates observed among females, despite lower overall infection rates, suggest possible differences in disease progression, healthcare access, or sociocultural factors influencing treatment‐seeking behaviour (Departamento de Epidemiología. Subsecretaría de Salud Pública. Ministerio de Salud. Gobierno de Chile 2021). This finding is consistent with that of Armien et al., whose 20‐year surveillance study in Panama also reported a slightly higher fatality rate in adult females than in adult males (Armién et al. 2023). That study, however, described a substantially lower overall case‐fatality rate than observed in Chile or the United States, likely because Panamanian cases are caused by Choclo orthohantavirus, which produces a considerably milder clinical profile, with only a small fraction of infections progressing to full HCPS (Armién et al. 2023). Readers should be aware that our findings on sex and age are restricted to hospitalized patients and correspond to associations within a selected clinical population for whom severity‐at‐admission data were unavailable; they cannot therefore be generalized to the entire population of HCPS cases.

Indigenous populations were disproportionately affected, with higher case‐fatality ratios compared to other groups. Systemic health inequities, including delayed healthcare access, underdiagnosis, comorbidities and cultural barriers to care, may underlie these disparities. Similar patterns have been reported in other zoonotic and infectious diseases affecting indigenous peoples, highlighting the need for tailored public health strategies (Butler et al. 2001; Millalen et al. 2020).

Our results emphasize the need for integrated One Health approaches to mitigate hantavirus risks. Surveillance systems should prioritize high‐risk districts, particularly during summer and incorporate wildfire monitoring to predict and prevent outbreak surges. Public health campaigns must address behavioural factors, educating communities about avoiding rodent exposure, particularly in wildfire‐affected areas (Holz and Palma 2012; Jaksic and Lima 2003). Efforts to reduce HCPS fatality rates should target healthcare disparities. Expanding access to early diagnostic tools and advanced care, including ECMO in tertiary hospitals, could improve outcomes for vulnerable populations (Departamento de Epidemiología. Ministerio de Salud. Gobierno de Chile 2021). At present, many patients must be transported to hospitals with ECMO capability, creating a referral delay that is likely to adversely affect clinical outcomes. While the 2017 recommendation for early hospitalization and transfer to ECMO‐capable centres represents an important public health advance, its effective implementation, the timeliness of referral, and the training of clinical teams are distinct operational dimensions that this study cannot evaluate and that represent a relevant area for future research. Specific interventions for indigenous groups should address both cultural and logistical barriers to healthcare access, ensuring timely treatment and preventive care (Millalen et al. 2020).

While this study provides robust evidence, several questions remain unanswered. Longitudinal studies examining the temporal dynamics of hantavirus risks in relation to environmental and sociocultural changes are warranted. High‐resolution spatial analyses could identify localized risk clusters and inform targeted interventions. Additionally, investigating the biological and sociocultural mechanisms behind the observed gender differences in fatality rates could guide more equitable clinical practices (Departamento de Epidemiología. Subsecretaría de Salud Pública. Ministerio de Salud. Gobierno de Chile 2021). The influence of climate change on hantavirus epidemiology deserves further exploration. Shifting rainfall patterns, temperature changes and increased wildfire frequency may alter rodent population dynamics and disease transmission (Martinez‐Valdebenito et al. 2014; Ecke et al. 2019) pathways, necessitating adaptive public health strategies. Several limitations of this study should be pointed out. First, for the ecological study, several unmeasured variables could be acting as confounders for the described associations, including rainfall patterns, plant growth and rodent abundance. We did not have access to high‐quality data on those variables and it rests on future studies to assess their possible association with both HCPS notifications and the modelled risk predictors. Second, the ecological outcome is notifications rather than directly observed infection incidence. Although notifications serve as a broadly adequate proxy, they are liable to carry biases related to care‐seeking behaviour, diagnostic access, surveillance sensitivity and reporting efficiency. Importantly, seroprevalence data from endemic areas of Chile, where IgG seroprevalence against ANDV reached only 2.5% in rural populations (Castillo et al. 2002), suggest that most individuals infected with hantavirus either present to a health facility due to symptoms or do not survive to be counted, meaning that notification data likely captures the clinically apparent fraction of the infection burden reasonably well, while oligosymptomatic or asymptomatic cases remain systematically underrepresented. Third, the ecological study carries the risk of ecological fallacy: the risk predictors we identified are applicable strictly at the district level, and they should not be interpreted as risk predictors for individual infections or positive diagnostic tests. Fourth, regarding the selection of individuals for the hospitalization study, because this study includes only hospitalized patients, it is possible for bias to be introduced by the criteria through which patients are derived to hospitals. This is especially relevant in this study given the 2017 change in guidelines. Even though we added a variable to control for this potential bias, there remains a risk of bias in patient selection during the study. Fifth, the DEIS data does not include information of severity‐at‐admission, nor other relevant severity considerations such as ICU admission or ventilatory support. This can potentially introduce biases in the prognostic interpretations as there is no normalized severity baseline, as noted by a previous systematic review (Tortosa et al. 2026). Readers should thus be aware that the risk predictors we identified might be influenced by the severity‐at‐admission. Sixth, length of hospitalization and number of surgical interventions are not baseline variables but intermediate variables that emerge during hospitalization. Their association with in‐hospital mortality should not be interpreted as causal, but as correlates reflecting disease severity and clinical trajectory. In particular, ‘surgical interventions’ is a broad and heterogeneous category that likely encompasses ECMO cannula insertion and other advanced life‐support procedures, as well as interventions required to manage complications arising during or after the acute phase. Their association with in‐hospital death should therefore be read as a marker of greater clinical complexity, not as an adverse effect of the procedures themselves. Seventh, even though we have discussed the use of ECMO as an advanced care option that is likely to improve patient outcomes, the current study does not have a means to isolate its impact from the potentially higher risk correlates associated with additional surgical interventions. A future study that assesses the isolated impact of ECMO would be highly desirable. In conclusion, this study underscores the complexity of hantavirus epidemiology, shaped by ecological, demographic and clinical factors. Addressing these challenges requires a multidisciplinary approach, integrating environmental management, healthcare equity and community engagement. By advancing surveillance and targeted interventions, these findings can contribute to reducing the burden of HCPS in Chile and beyond.

## Author Contributions

B.D.V. designed and performed the computational experiments, interpreted the results and wrote the initial manuscript. N.A. conducted all spatial analyses, interpreted the results and reviewed the manuscript. M.F. and F.O.M. conceived the study, interpreted the results and reviewed the manuscript. F.S.‐R., C.M.‐V., C.H. and F.C.‐B. interpreted the results and reviewed the manuscript. All authors read and approved the final manuscript.

## Funding

This work was supported by Agencia Nacional de Investigación y Desarrollo, 1211825, 21230036, 21221163.

## Disclosure

Preliminary results from this study were presented at the Primeras Jornadas de Ciencia de Datos y Salud Pública, Escuela de Salud Salud Pública and Universidad de Chile in 16–18 january, 2024.

## Ethics Statement

This study used exclusively anonymized, publicly available administrative datasets obtained from the Chilean Ministry of Health (MINSAL) notification database and the Chilean Department of Health Statistics and Information (DEIS) hospitalization database. No individual patient contact was made, no biological samples were collected, and no experimental procedures were performed on human subjects or animals. In accordance with Chilean regulations and international guidelines for the use of anonymized secondary data, formal ethical approval was not required for this study. All data were handled in strict accordance with applicable data protection and confidentiality standards.

## Conflicts of Interest

The authors declare no conflicts of interest.

## Supporting information


**Figure S1:** HCPS Notifications Time Series Decomposition.
**Figure S2:** ACF and PACF charts for the HCPS notifications time series. A: ACF. B: PACF.
**Figure S3:** HCPS Hospitalizations Time Series Decomposition.
**Figure S4:** ACF and PACF charts for the HCPS hospitalization time series. A: ACF. B: PACF.
**Figure S5:** HCPS notifications time series decomposition showing the distribution of cases by month throughout the study period. The number on the *x*‐axis indicates the calendar month from January (1) to December (12).
**Figure S6:** HCPS hospitalizations time series decomposition showing the distribution of cases by month throughout the study period. The number on the *x*‐axis indicates the calendar month from January (1) to December (12).
**Table S1:** Coefficients, standard errors, statistics, *p*‐values and confidence intervals for the final negative binomial regression with mixed effects model.
**Table S2:** Coefficients, standard errors, statistics, *p*‐values and confidence intervals for the final logistic regression with mixed effects model.

## Data Availability

The data used to support the findings of this study, and all analyses can be reproduced with scripts available in the following GitHub repository: https://github.com/unasaluduc.

## References

[zph70084-bib-0001] Armién, B. , C. Muñoz , H. Cedeño , et al. 2023. “Hantavirus in Panama: Twenty Years of Epidemiological Surveillance Experience.” Viruses 15: 1395. 10.3390/v15061395. PMC10304491 37376694

[zph70084-bib-0002] Belmar‐Lucero, S. , P. Godoy , M. Ferrés , P. Vial , and R. E. Palma . 2009. “Range Expansion of *Oligoryzomys longicaudatus* (Rodentia, Sigmodontinae) in Patagonian Chile, and First Record of Hantavirus in the Region.” Revista Chilena de Historia Natural 82: 265–275. 10.4067/S0716-078X2009000200008.

[zph70084-bib-0003] Bonilla‐Aldana, D. K. , J. A. Suárez , C. Franco‐Paredes , et al. 2019. “Brazil Burning! What Is the Potential Impact of the Amazon Wildfires on Vector‐Borne and Zoonotic Emerging Diseases? – A Statement From an International Experts Meeting.” Travel Medicine and Infectious Disease 31: 101474. 10.1016/j.tmaid.2019.101474. 31494225

[zph70084-bib-0004] Butler, J. C. , S. Crengle , J. E. Cheek , et al. 2001. “Emerging Infectious Diseases Among Indigenous Peoples.” Emerging Infectious Diseases 7: 554–555. 10.3201/eid0707.017732. PMC2631850 11485670

[zph70084-bib-0005] Butler, J. C. , and C. J. Peters . 1994. “Hantaviruses and Hantavirus Pulmonary Syndrome.” Clinical Infectious Diseases 19: 387–395. 10.1093/clinids/19.3.387. 7811854

[zph70084-bib-0006] Castillo, C. , L. Sanhueza , M. Täger , S. Muñoz , G. Ossa , and P. Vial . 2002. “Seroprevalence of Antibodies Against Hantavirus in 10 Communities of the IX Region of Chile Where Hantavirus Infection Were Diagnosed.” Revista Médica de Chile 130, no. 3: 251–258. 10.4067/S0034-98872002000300002. 12043366

[zph70084-bib-0007] Centers for Disease Control and Prevention . 2021. Hantavirus. CDC ‐ Hantavirus.

[zph70084-bib-0008] Departamento de Epidemiología. Ministerio de Salud. Gobierno de Chile . 2021. “MINUTA. TEMA:Situación de Hantavirus 2021.”

[zph70084-bib-0009] Departamento de Epidemiología. Subsecretaría de Salud Pública. Ministerio de Salud. Gobierno de Chile . 2021. Informe EPIDEMIOLÓGICO ANUAL Hantavirus.

[zph70084-bib-0010] Douglas, K. O. , K. Payne , G. Sabino‐Santos , and J. Agard . 2021. “Influence of Climatic Factors on Human Hantavirus Infections in Latin America and the Caribbean: A Systematic Review.” Pathogens 11: 15. 10.3390/pathogens11010015. PMC8778283 35055965

[zph70084-bib-0011] Ecke, F. , S. A. Nematollahi Mahani , M. Evander , B. Hörnfeldt , and H. Khalil . 2019. “Wildfire‐Induced Short‐Term Changes in a Small Mammal Community Increase Prevalence of a Zoonotic Pathogen?” Ecology and Evolution 9: 12459–12470. 10.1002/ece3.5688. PMC6875567 31788190

[zph70084-bib-0012] Ferrés, M. , C. Sandoval , I. Delgado , V. Sotomayor , A. Olea , and P. A. Vial . 2010. “Hantavirosis: Clinical and Epidemiological Characteristics of Pediatric Patients in Chile.” Revista Chilena de Infectología 27, no. 1: 52–59. 10.4067/S0716-10182010000100009. 20140316

[zph70084-bib-0013] Ferrés, M. , P. Vial , C. Marco , et al. 2007. “Prospective Evaluation of Household Contacts of Persons With Hantavirus Cardiopulmonary Syndrome in Chile.” Journal of Infectious Diseases 195: 1563–1571. 10.1086/516786. 17471425

[zph70084-bib-0014] Fritz, R. , F. Arancibia , R. Gutiérrez‐Arias , C. Sepulveda , C. Guerra , and C. Paulsen . 2023. “Risk Factors Associated With Hantavirus Cardiopulmonary Syndrome in Chile. In Proceedings of the Acute Critical Care”. European Respiratory Society.

[zph70084-bib-0015] Gutiérrez‐Jara, J. P. , M. T. Muñoz‐Quezada , F. Córdova‐Lepe , and A. Silva‐Guzmán . 2023. “Mathematical Model of the Spread of Hantavirus Infection.” Pathogens 12: 1147. 10.3390/pathogens12091147. PMC10536976 37764955

[zph70084-bib-0016] Holz, A. , and R. E. Palma . 2012. “Floraciones de Bambúes En Chile y Argentina: Actual Floración Masiva Del Colihue, Historia Natural y Riesgos Asociados.” Revista Bosque Nativo 50: 40–46.

[zph70084-bib-0017] Instituto Nacional de Estadísticas . 2021. “Censo de Población y Vivienda. Censo de Población y Vivienda.”

[zph70084-bib-0018] Jaksic, F. M. , and M. Lima . 2003. “Myths and Facts on Ratadas: Bamboo Blooms, Rainfall Peaks and Rodent Outbreaks in South America.” Austral Ecology 28: 237–251. 10.1046/j.1442-9993.2003.01271.x.

[zph70084-bib-0019] Knipe, D. M. , and P. M. Howley . 2013. Fields Virology. 6th ed. Wolters Kluwer health ‐ Lippincott Williams & Wilkins.

[zph70084-bib-0020] Martinez‐Valdebenito, C. , M. Calvo , C. Vial , et al. 2014. “Person‐To‐Person Household and Nosocomial Transmission of Andes Hantavirus, Southern Chile, 2011.” Emerging Infectious Diseases 20: 1637–1644. 10.3201/eid2010.140353. PMC4193174 25272189

[zph70084-bib-0021] Medina, R. A. , F. Torres‐Perez , H. Galeno , et al. 2009. “Ecology, Genetic Diversity, and Phylogeographic Structure of Andes Virus in Humans and Rodents in Chile.” Journal of Virology 83: 2446–2459. 10.1128/JVI.01057-08. PMC2648280 19116256

[zph70084-bib-0022] Millalen, P. , H. Nahuelpan , A. Hofflinger , and E. Martinez . 2020. “COVID‐19 and Indigenous Peoples in Chile: Vulnerability to Contagion and Mortality.” AlterNative: An International Journal of Indigenous Peoples 16: 399–402. 10.1177/1177180120967958.

[zph70084-bib-0023] Ministerio de Salud . 2013. GUÍA CLÍNICA de PREVENCIÓN, DIAGNÓSTICO Y TRATAMIENTO Del SÍNDROME CARDIOPULMONAR Por Hantavirus.

[zph70084-bib-0024] Ministerio de Salud . 2018. “Síndrome Cardiopulmonar Por Hantavirus. Síndrome cardiopulmonar por hantavirus ‐ EPI ‐ Departamento de Epidemiología.”

[zph70084-bib-0025] Nsoesie, E. O. , S. R. Mekaru , N. Ramakrishnan , M. V. Marathe , and J. S. Brownstein . 2014. “Modeling to Predict Cases of Hantavirus Pulmonary Syndrome in Chile.” PLoS Neglected Tropical Diseases 8: e2779. 10.1371/journal.pntd.0002779. PMC3998931 24763320

[zph70084-bib-0026] Palma, R. E. , D. Boric‐Bargetto , F. Torres‐Pérez , C. E. Hernández , and T. L. Yates . 2012. “Glaciation Effects on the Phylogeographic Structure of *Oligoryzomys longicaudatus* (Rodentia: Sigmodontinae) in the Southern Andes.” PLoS One 7: e32206. 10.1371/journal.pone.0032206. PMC3291571 22396751

[zph70084-bib-0027] Palma, R. E. , J. J. Polop , R. D. Owen , and J. N. Mills . 2012. “Ecology of Rodent‐Associated Hantaviruses in the Southern Cone of South America: Argentina, Chile, Paraguay, and Uruguay.” Journal of Wildlife Diseases 48: 267–281. 10.7589/0090-3558-48.2.267. 22493103

[zph70084-bib-0028] Pascoe, E. L. , B. T. Plourde , A. M. Lopéz‐Perez , and J. E. Foley . 2020. “Response of Small Mammal and Tick Communities to a Catastrophic Wildfire and Implications for Tick‐Borne Pathogens.” Journal of Vector Ecology 45: 269–284. 10.1111/jvec.12398. 33207067

[zph70084-bib-0029] R Core Team . 2023. R: A Language and Environment for Statistical Computing. R Foundation for Statistical Computing: Vienna, Austria.

[zph70084-bib-0030] Reyes, R. , K. Yohannessen , S. Ayala , and M. Canals . 2019. “Estimaciones de La Distribución Espacial Del Riesgo Relativo de Mortalidad Por Las Principales Zoonosis En Chile: Enfermedad de Chagas, Hidatidosis, Síndrome Cardiopulmonar Por Hantavirus y Leptospirosis.” Revista Chilena de Infectología 36: 599–606. 10.4067/S0716-10182019000500599. 31859801

[zph70084-bib-0031] Riccò, M. , S. Peruzzi , S. Ranzieri , and N. Magnavita . 2021. “Occupational Hantavirus Infections in Agricultural and Forestry Workers: A Systematic Review and Metanalysis.” Viruses 13: 2150. 10.3390/v13112150. PMC8621010 34834957

[zph70084-bib-0032] Sotomayor P, E. U. V. , and X. Aguilera S . 2000. “Epidemiología de La Infección Humana Por Hantavirus En Chile.” Revista Chilena de Infectología 17: 265–275. 10.4067/S0716-10182000000300006.

[zph70084-bib-0033] Täger, M. F. , P. C. Vial , C. H. Castillo , P. M. Godoy , B. Hjelle , and M. G. Ferrés . 2003. “Hantavirus Prevalence in the IX Region of Chile.” Emerging Infectious Diseases 9: 827–832. 10.3201/eid0907.020587. PMC3023426 12890323

[zph70084-bib-0034] Thorp, L. , L. Fullerton , A. Whitesell , and W. Dehority . 2023. “Hantavirus Pulmonary Syndrome: 1993–2018.” Pediatrics 151: e2022059352. 10.1542/peds.2022-059352. 36855865

[zph70084-bib-0035] Torres‐Pérez, F. , R. E. Palma , D. Boric‐Bargetto , et al. 2019. “A 19 Year Analysis of Small Mammals Associated With Human Hantavirus Cases in Chile.” Viruses 11: 848. 10.3390/v11090848. PMC6784195 31547341

[zph70084-bib-0036] Tortosa, F. , M. A. Ragusa , I. Neumann , et al. 2026. “Prognostic Factors for Mortality in Patients Infected With New World Hantaviruses: A Systematic Review and Meta‐Analysis.” BMJ Open 16: e096313. 10.1136/bmjopen-2024-096313. PMC12853536 41592833

[zph70084-bib-0037] Vial, P. A. , M. Ferrés , C. Vial , et al. 2023. “Hantavirus in Humans: A Review of Clinical Aspects and Management.” Lancet Infectious Diseases 23: e371–e382. 10.1016/S1473-3099(23)00128-7. 37105214

