# Supplementary material for: Ecological and Individual‐Level Factors Associated With Hantavirus Cardiopulmonary Syndrome Notifications and in‐Hospital Mortality Among Hospitalized Patients in Chile, 2005–2022
Source: Zoonoses Public Health. 2026 Aug 16;73(7):644–54. doi: 10.1111/zph.70084 (PMC13633442; doi:10.1111/zph.70084)
Supplement: Supplementary file 1 [file ZPH-73-644-s001.docx]

**Supplementary Materials for:**

**Ecological and Individual-Level Factors Associated with Hantavirus Cardiopulmonary Syndrome Notifications and In-Hospital Mortality among Hospitalized Patients in Chile, 2005–2022**

Benjamín Diethelm-Varela^1,2^, Nicolhole Atero^2^, Fernanda Sánchez^3^, Constanza Martínez-Valdebenito^4^, Carolina Henríquez^4^, Francisca Córdova-Bührle^5^, Marcela Ferrés^4*^, Fernando O. Mardones^6*^

^1^ Escuela de Gobierno. Pontificia Universidad Católica de Chile, Santiago, Chile.

^2^ SENTINET: Surveillance, Epidemiology, and New Technologies for Infectious Emerging Threats, Av. Vicuña Mackenna 4860, Macul, Santiago, Región Metropolitana 7820436, Chile

^3^ Escuela de Medicina Veterinaria, Pontificia Universidad Católica de Chile, Santiago, Chile.

^4^ Departamento de Enfermedades Infecciosas e Inmunología Pediátrica, Escuela de Medicina, Pontificia Universidad Católica de Chile, Santiago, Chile.

^5^ Facultad de Ciencias Veterinarias y Pecuarias (FAVET), Universidad de Chile, Santiago, Chile.

^6^ Division of Global Agriculture and Food Systems, the Royal (Dick) School of Veterinary Studies, the University of Edinburgh, Edinburgh, UK.

* Correspondence to: fmardone@ed.ac.uk, mferres@uc.cl

# Supplementary Information

Additive time series models were constructed for HCPS notifications and hospitalizations. Graphs of the observed time series along with the trend, seasonal, and random components obtained from the decomposition are available below (Figure S1 and Figure S3 for notifications and hospitalizations, respectively).

For the notifications time series, the Augmented Dickey-Fuller test of stationarity yielded a test statistic of -7.94 (p = 0.01). The null hypothesis of non-stationarity is rejected, and thus the alternative hypothesis that the data is stationary is accepted. The Mann-Kendall test of monotonic trend yielded a test statistic of -1.8 (p = 0.08). This result indicates that there is not enough evidence to assert that a monotonic trend exists in the data. The Pettitt test of single change point yielded a test statistic of 2886 (p = 0.01). The result supports the alternative hypothesis that the series is two-sided, that is, that there is a change point in the series. After removing the trend and seasonal components from the time series, we plotted the ACF and PACF, which indicated that no significant autocorrelation is present, suggesting that HCPS events are independent. The ACF and PACF plots are available in the supplementary materials (Figure S2).

For the hospitalizations time series, The Augmented Dickey-Fuller test of stationarity yielded a test statistic of -4.92 (p = 0.01). The null hypothesis of non-stationarity is rejected, and thus the alternative hypothesis that the data is stationary is accepted. The Mann-Kendall test of monotonic trend yielded a test statistic of 0.7 (p = 0.49). This result indicates that there is not enough evidence to assert that a monotonic trend exists in the data. The Pettitt test of single change point yielded a test statistic of 865 (p = 0.29). The result does not support the alternative hypothesis that the series is two-sided, and thus it can’t be asserted that a change point exists in the series. After removing the trend and seasonal components from the time series, we plotted the ACF and PACF, which indicated that no significant autocorrelation is present, suggesting that HCPS events are independent. The ACF and PACF plots are available in the supplementary materials (Figure S4).

Given the seasonality we observed in the notifications and hospitalizations data, we decided to further the time series decomposition analysis. The distributions of cases by month throughout the study period (Figure S5 and Figure S5 for notifications and hospitalizations respectively) corroborate our previous observation of seasonality, showing a marked, significant increase of cases during the summer months of January, February, and March for both notifications and hospitalizations (F = 13.42, p < 0.001 for notifications, and F = 5.67, p < 0.001 for hospitalizations).

**Figure S1.** HCPS Notifications Time Series Decomposition

**Figure S2.** ACF and PACF charts for the HCPS notifications time series. A: ACF. B: PACF

**Figure S3.** HCPS Hospitalizations Time Series Decomposition

**Figure S4.** ACF and PACF charts for the HCPS hospitalization time series. A: ACF. B: PACF

**Figure S5.** HCPS notifications time series decomposition showing the distribution of cases by month throughout the study period. The number on the x-axis indicates the calendar month from January (1) to December (12)

**Figure S6.** HCPS hospitalizations time series decomposition showing the distribution of cases by month throughout the study period. The number on the x-axis indicates the calendar month from January (1) to December (12)

**Table S1.** Coefficients, standard errors, statistics, p-values, and confidence intervals for the final negative binomial regression with mixed effects model

| Type of Effect | Grouping variable | Term | Point Estimate | Standard Error | Statistic | p-value | 95% CI lower bound | 95% CI higher bound |
| --- | --- | --- | --- | --- | --- | --- | --- | --- |
| fixed | NA | Intercept | -14.55 | 0.24 | -61.63 | 0.00 | -15.02 | -14.09 |
| fixed | NA | Year | -0.08 | 0.04 | -2.18 | 0.03 | -0.15 | -0.01 |
| fixed | NA | Burned Surface/district Area | 0.13 | 0.01 | 11.14 | 0.00 | 0.10 | 0.15 |
| fixed | NA | Urbanization Proportion | -0.58 | 0.06 | -9.17 | 0.00 | -0.70 | -0.45 |
| fixed | NA | Males per 100,000 inh. | 0.35 | 0.12 | 2.79 | 0.01 | 0.10 | 0.59 |
| fixed | NA | Density | -0.23 | 0.08 | -2.70 | 0.01 | -0.39 | -0.06 |
| fixed | NA | Season - Fall | -0.72 | 0.09 | -7.80 | 0.00 | -0.90 | -0.54 |
| fixed | NA | Season - Winter | -1.21 | 0.11 | -11.18 | 0.00 | -1.42 | -1.00 |
| fixed | NA | Season - Spring | -0.91 | 0.10 | -9.26 | 0.00 | -1.10 | -0.71 |
| random | Province | Random Effect | 1.40 | NA | NA | NA | NA | NA |

**Table S2.** Coefficients, standard errors, statistics, p-values, and confidence intervals for the final logistic regression with mixed effects model

| Type of Effect | Grouping variable | Term | Point Estimate | Standard Error | Statistic | p-value | 95% CI lower bound | 95% CI higher bound |
| --- | --- | --- | --- | --- | --- | --- | --- | --- |
| fixed | NA | Intercept | -2.70 | 0.34 | -7.89 | 0.00 | -3.37 | -2.03 |
| fixed | NA | Sex (reference: male) | 0.71 | 0.32 | 2.18 | 0.03 | 0.07 | 1.34 |
| fixed | NA | Age (years) | 0.78 | 0.15 | 5.14 | 0.00 | 0.48 | 1.08 |
| fixed | NA | Indigenous Group (reference: does not belong) | 0.73 | 0.37 | 1.99 | 0.05 | 0.01 | 1.45 |
| fixed | NA | Length of Hospitalization (days) | -2.04 | 0.32 | -6.31 | 0.00 | -2.68 | -1.41 |
| fixed | NA | Number of Surgical Interventions | 0.83 | 0.15 | 5.57 | 0.00 | 0.54 | 1.12 |
| random | Hospital | Random Effect | 0.51 | NA | NA | NA | NA | NA |

:::
